# Effect of earthworm *Eisenia fetida* epidermal mucus on the vitality and pathogenicity of *Beauveria bassiana*

**DOI:** 10.1038/s41598-021-92694-y

**Published:** 2021-07-06

**Authors:** Xibei Zhou, Wulong Liang, Yanfeng Zhang, Zhumei Ren, Yingping Xie

**Affiliations:** School of Life Science, Shanxi University, 92 Wucheng Rd, Taiyuan, 030006 Shanxi China

**Keywords:** Microbiology, Zoology

## Abstract

*Beauveria bassiana* is one of the most widely studied and used entomopathogenic fungus as biopesticide. In the biological control of pests, *B. bassiana* will persist in the soil after application, and will inevitably contact with earthworms, especially the epigeic earthworm species*.* So, what are the effects of earthworm and its epidermal mucus on the activity of *B. bassiana*? We employed the epigeic earthworm *Eisenia fetida, B. bassiana* TST05 strain, and the insect *Atrijuglans hetaohei* mature larvae to study the impact of earthworm epidermal mucus on the vitality and pathogenicity of *B. bassiana* to insect*.* Methods included scanning electron microscope observation, detection of spore germination, fungal extracellular enzyme activity, and infection testing to *A. hetaohei*. The results showed that the *B. bassiana* spores may attach to the cuticle of *E. fetida* but they could be covered by the epidermal mucus and became rough and shrunken. After treatment with the epidermal mucus, the spore germination and extracellular enzymes of *B. bassiana* was significantly inhibited. Inoculation of *A. hetaohei* larvae with a mixture of *B. bassiana* and mucus showed that the mucus could reduce the pathogenicity of *B. bassiana* to the insect, resulting in a slower disease course and lower mortality. It was concluded that the epidermal mucus of the earthworm *E. fetida* can inhibit the activity of *B. bassiana*, as well as the infectivity and pathogenicity of fungus to target insects. However, after treatment with epidermal mucus the surviving *B. bassiana* still had certain infectivity to insects. This is of great significance for the application of *B. bassiana* in biological control of pests.

## Introduction

*Beauveria bassiana*, as a broad-spectrum entomopathogenic fungus, can infect more than 700 species of insects in 149 families of 15 orders and more than ten species of Acarina. It is also one of the most widely studied and used entomopathogenic fungi^1,2^. When *B. bassiana* infects insects, spores can adhere to the insect body surface through hydrophobic interactions. The spores absorb moisture from the insect surface to germinate and produce germ tubes. A variety of extracellular enzymes, such as chitinase and protease, are secreted from the tip of germ tubes, which can dissolve chitin and protein in the insect cuticle. The germ tubes extend continuously and penetrate into the insect cuticle and then enter the insect coelom. With the propagation of blastospores, the hyphae of *B. bassiana* penetrate the cuticle of insect and invade the blood cavity, infecting blood cells and internal organs. Arthrospores germinated and produced large amounts of toxins. When spores and hyphae develop to a certain number, the spores begin to decline and the hyphae increase gradually. The hyphae invade the body fat, digestive tract, Malpighian tubules, ganglion and trachea, as well as other tissues and organs, and can destroy the cells and lead to death of the insect^3,4^.

Fungus *B. bassiana* can persist in soil and play an important role in the regulation of insect populations in soil under natural conditions^5^. Fungus *B. bassiana* is widely used as a biopesticide to control pests in farmland, orchards, vegetable fields and forests^6–8^. After application, *B. bassiana* can enter the soil and persist for a long time. It plays an important role in the control of pests^9,10^.

Earthworms are ubiquitous in farmland, orchards, vegetable gardens and woodlands and play important ecological functions in soil, such as loosening soil, improving aggregate structure, regulating soil acidity, decomposing organic matter in the soil, and improving soil fertility^11,12^.

Earthworms can secrete mucus from their epidermal secreting cells. The epidermal mucus together with coelomic fluid discharged from the dorsal pores forms a mucus layer over their body surface. The earthworm mucus is a water-soluble mixture of low molecular weight carbohydrates, aminoacids, glycosides and glycoproteins. Among them, mucus proteins are the basic components of innate immunity^13–16^. The epidermal mucus can keep the skin moist, ensure normal breathing, and play a lubricating role in the movement of earthworms. Epidermal mucus is also an important immune barrier for earthworms against pathogenic microorganisms in soil^17,18^. Earthworms can affect the species structure in soil by interacting with other organisms. It has been reported that earthworm epidermal mucus can inhibit some bacteria and affect the activities and abundance of bacteria in soil^19,20^. Mucus also has an obvious effect on the physiological function of several nematodes inhabiting soil and can regulate the quantity and distribution of these nematodes^10,21,22^.

However, it has not been reported whether the earthworm epidermal mucus affects the activity of *B. bassiana*. In the biological control of pests, *B. bassiana* will persist in the soil after application, and will inevitably contact with earthworms, especially the epigeic earthworm species, e.g. *E. fetida.* So, what are the effects of earthworm and its epidermal mucus on the activity of *B. bassiana*? We hypothesized that the presence of the earthworm epidermal mucus might have an antifungal effect on *B. bassiana*, thereby reducing their fitness. The aim of the present study was to investigate the impact of the earthworm epidermal mucus on *B. bassiana* biological control efficacy.

We employed a model system composed of earthworm *E. fetida* (Savigny) (Opisthopora: Lumbricidae), *B. bassiana* strain TST05 and the mature larvae of *A. hetaohei* Yang (Lepidoptera: Heliodinidae). In which, *E. fetida* is an epigeic species, and is the most commonly species used for composting with widespread distribution^22^. The strain *B. bassiana* TST05 is a highly pathogenic strain that was originally isolated in 2009 by our laboratory from the naturally infected overwintering larvae of *Carposina sasakii* (Matsumura) (Lepidoptera: Carposinidae) in the soil of apple orchards in Xiangfen County, Shanxi Province, China. On the basis of morphological identification, the strain was identified by molecular technology. After BLAST comparison, the homology of TST05 with *B. bassiana* (Accession No. JN713138, HQ444271) and *Cordyceps bassiana* (Accession No. AJ564808) in GenBank was 99%, and the support rate was 100% on the same branch of the evolutionary tree^23^. Therefore, the TST05 strain was identified as *B. bassiana*. The biological characteristics of the *B. bassiana* TST05 strain, pathogenicity to host insects, persistence in soil, and compatibility with chemical insecticides were studied in previous researches^24–26^. At the same time, the strain was deposited in the China General Microbial Species Conservation and Management Center (Beijing, China) under storage number CGMCC4526. The mature larvae of *A. hetaohei* Yang (Lepidoptera: Heliodinidae) were selected as the target insects infected by *B. bassiana* strain TST05. It is an important pest of walnut fruit in northern China. The insect produces one generation a year, and its larvae bore into walnut fruit to feed on the pulp, which makes the walnut fruit lose its commercial value. The newly hatched larvae bore into the fruit in May and develop to maturity in late August. They drill out of the fruit and fall into the soil layer to live through the winter and cocoon and pupate. In the early summer of the next year, the adults emerge and bore out of the soil and then mate and lay their eggs in walnut trees. Under natural conditions, larvae are often infected by entomogenous fungi such as *B. bassiana* in soil. The biological control of *A. hetaohei* using *B. bassiana* and other entomopathogenic fungi has attracted much attention^27^. An infection test of *B. bassiana* strain TST05 on *A. hetaohei* and the application of a strain preparation in walnut orchard soil were carried out in prior study to control the mature larvae, and high infection mortality was achieved^28^. Therefore, the mature larvae of *A. hetaohei* were selected as the target insects infected by *B. bassiana* strain TST05 in this study. In addition, after entering the soil, the mature larvae of *A. hetaohei* can exist together with earthworm *E. fetida* and *B. bassiana* TST05 strain for 8–9 months. Thus, it is a typical insect in the walnut orchard soil environment.

This study provides a reference for understanding the impact of earthworm epidermal mucus to *B. bassiana* in the activity and pathogenicity to the insects. It is significant for the application of *B. bassiana* as a biopesticide in the field.

## Results

### Attachment of *B. bassiana* to the body surface of *E. fetida*

**Figure 1:**
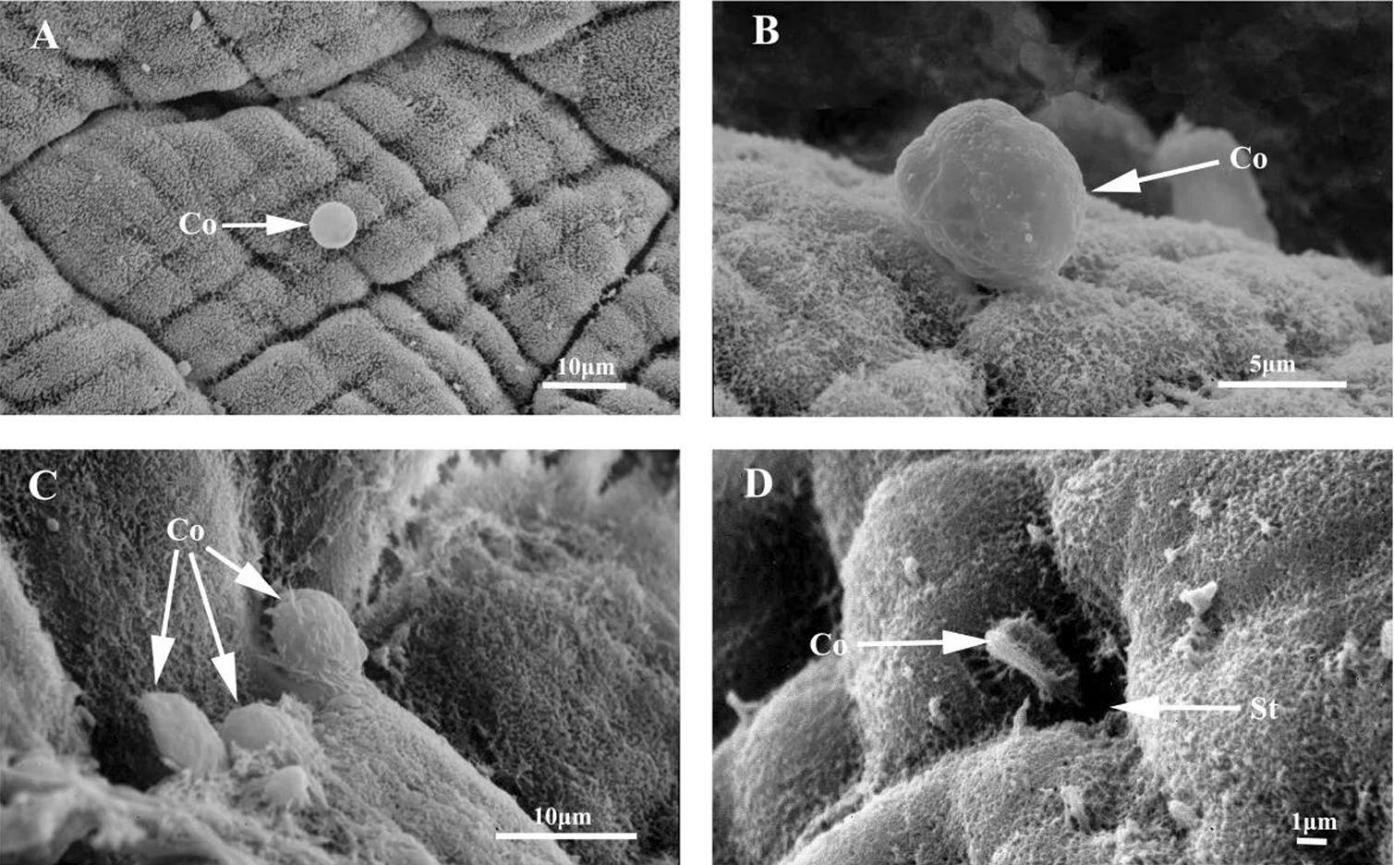
SEM photos of *B. bassiana* spores attachment to the body surface of the earthworm *E. foetida*. (**A**) The grid-like structures on the body segments of the earthworm *E. foetida* and the attached *B. bassiana* spores. (**B**) the attached *B. bassiana* spores were wrapped by the epidermis mucus of the earthworm *E. foetida*. (**C**) the attached *B. bassiana* spores changed into shrunk and shriveled. (**D**) the *B. bassiana* spores attached near the stomata and covered with the epidermis mucus of the earthworm *E. foetida*. Co: *B. bassiana* spores; St: Stomata of the earthworm *E. foetida*.

Under SEM, it was observed that *B. bassiana* spores often adhere to the intersegmental furrows between the segments, annulus on the segments, grid-like structures between the annuli, and those around the stomata of *E. fetida* (Fig. 1). As time progressed, the attached spores of *B. bassiana* exhibited obvious changes in morphology. After 1 h of incubation, the attached spores remained smooth, round and plump in shape (Fig. 1A). After 3 d, the spores changed to rough and uneven because of the earthworm epidermal mucus covering (Fig. 1B). After 7 d, the spores had shrunken and shriveled (Fig. 1C). Some spores were also observed near the stomata, and they were also covered with the earthworm epidermal mucus (Fig. 1D). In observation, although spore attachment, morphological change, and covering with the earthworm epidermal mucus were observed, spore germination was not seen on the body surface of the earthworms.

### Effect of *E. fetida* epidermal mucus on spore germination of *B. bassiana*

**Figure 2:**
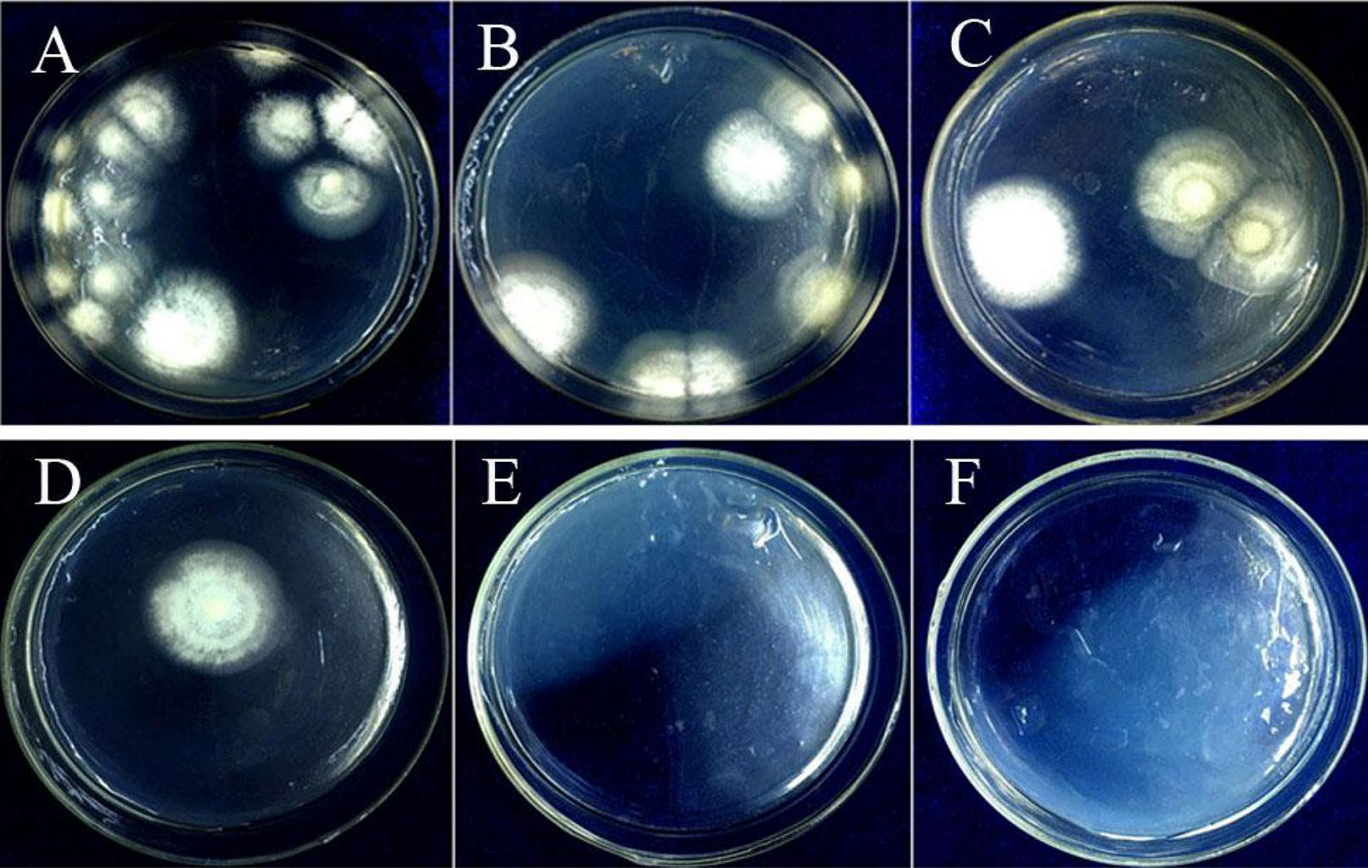
On the plate culture media, the comparison of the spore germination and colony growth of *B. bassiana* treated with the epidermis mucus of the earthworm *E. foetida* in different concentrations. (**A**): Control group, in which, before inoculation, the *B. bassiana* spores were not treated with the earthworm epidermis mucus. (**B–F**): Treatment groups, in which, before inoculation, the *B. bassiana* spores were treated with the earthworm epidermis mucus in the concentrations of 1.25 mg/mL (**B**), 2.50 mg/mL (**C**), 5.00 mg/mL (**D**), 10.00 mg/mL (**E**), and 20.00 mg/mL (**F**), respectively.

**Figure 3:**
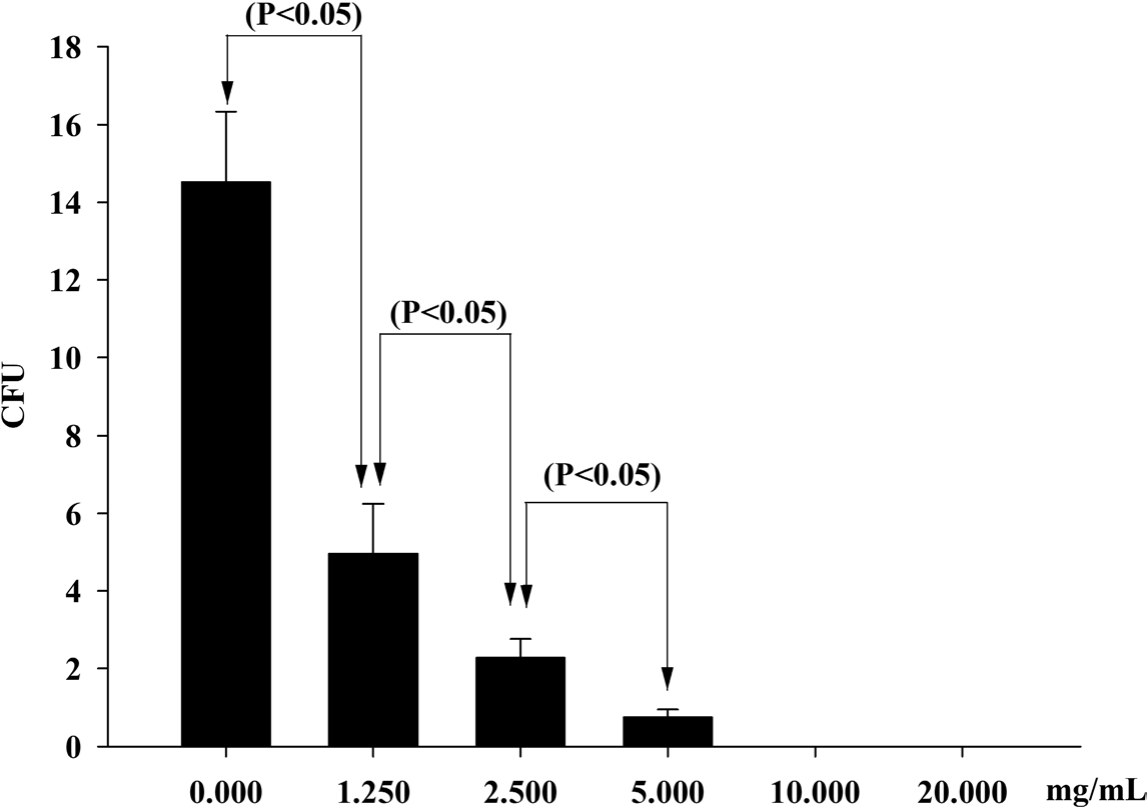
Statistical analysis of the colony quantities of *B. bassiana* treated with the epidermis mucus of the earthworm *E. foetida* in different concentrations.

After incubating with solutions of epidermal mucus, the colonies of *B. bassiana* exhibited obvious differences after treatment with the earthworm epidermal mucus (Fig. 2). In the control group that was not incubated with *E. fetida* epidermal mucus, *B. bassiana* grew considerably more colonies on the plate (Fig. 2A), with 14.20 colonies on average (Fig. 3). In contrast, less *B. bassiana* grew in the groups treated with *E. fetida* epidermal mucus. As the concentration of *E. fetida* epidermal mucus increased, the number of colonies gradually decreased (Fig. 2B–F). In total, an average of 5.60 fungal colonies were noted on the culture plates that were inoculated spores treated with 1.25 mg/mL *E. fetida* epidermal mucus solution, a decrease of 60.6% (Figs. 2B, 3). In comparison, the number of fungal colonies decreased to an average of 2.20 upon treatment with 2.50 mg/mL *E. fetida* epidermal mucus solution, a decrease of 84.5% (Figs. 2C, 3). The average number of colonies was only 0.40 upon treatment with 5.00 mg/mL epidermal mucus solution, a decrease of 97.2% (Figs. 2D, 3). An extreme change was observed upon treatment with 10.00 mg/mL and 20.00 mg/mL *E. fetida* epidermal mucus solutions. Specifically, no colonies grew on the culture plates (Figs. 2E,F, 3). Statistical analysis showed that the number of *B. bassiana* colonies significantly differed between the treatment and control groups. With the increase in the *E. fetida* epidermal mucus concentration, the number of colonies decreased significantly (Fig. 3). Therefore, the *E. fetida* epidermal mucus had a significant inhibitory effect on *B. bassiana* spore germination. This inhibitory effect was correlated with the concentration of *E. fetida* epidermal mucus. When the concentration of earthworm epidermal mucus was more than 10.00 mg/mL, the inhibitory effect on *B. bassiana* spores reached 100%. A significant Linear correlation was found between earthworm mucus concentration and fungal colony number as follows: Y = −2.5429X + 12.653 (R^2^ = 0.7222).

### Inhibitory effect of *E. fetida* epidermal mucus on extracellular enzyme activity of *B. bassiana*

**Figure 4:**
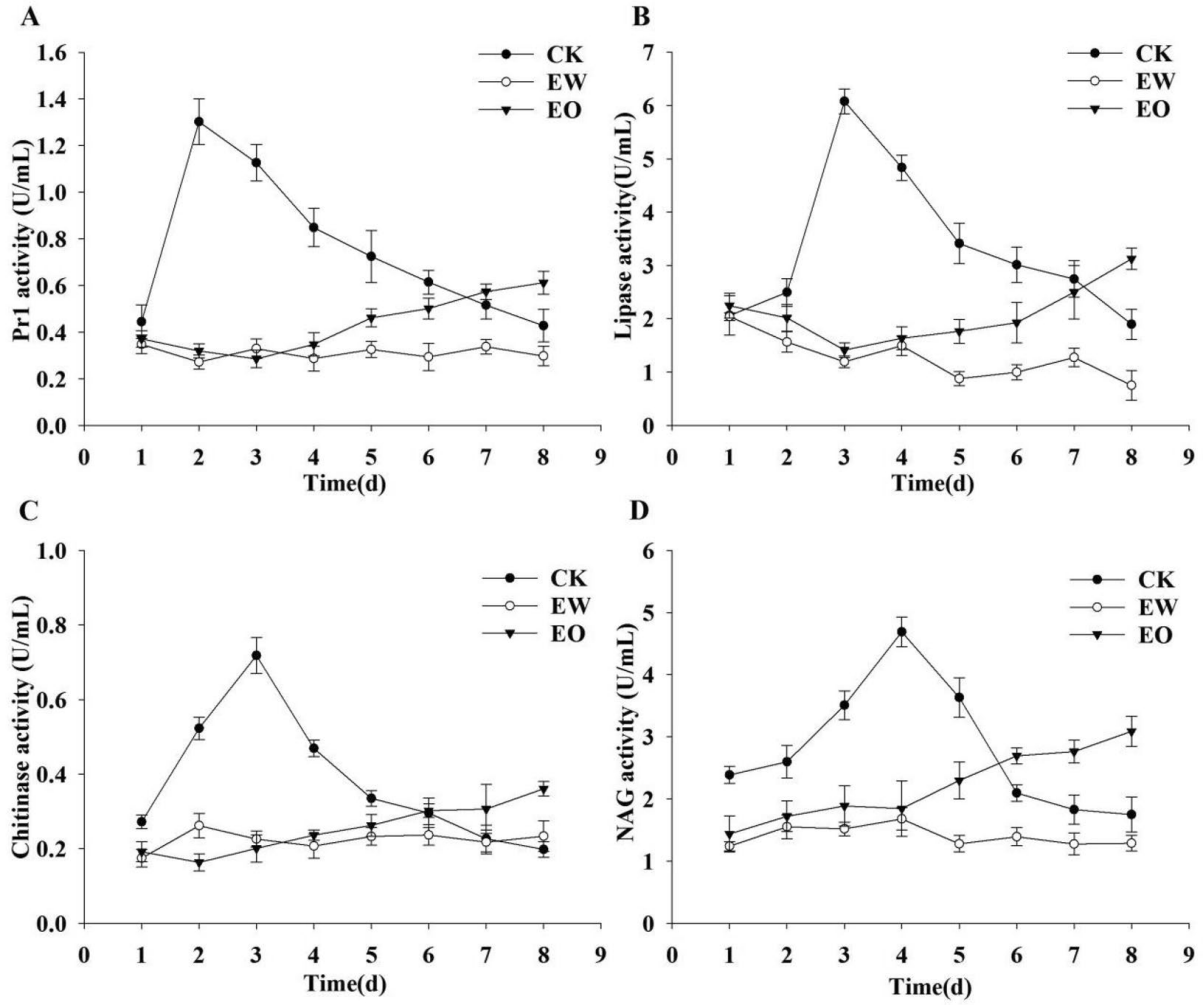
Comparison of the inhibitory effects of the epidermal mucus of the earthworm *E. foetida* on the 4 extracellular enzyme activities of *B. bassiana*. (**A**): subtilisin-like protease, (**B**): lipase, (**C**): chitinase, and (**D**): NAG. CK: the control group, no added the epidermal mucus of the earthworm *E. foetida* to the liquid medium. EW: treatment group, added the epidermal mucus of the earthworm *E. foetida* to the liquid medium once before inoculation and replenished the epidermal mucus every day during inoculation. EO: added the earthworm epidermis mucus to the liquid medium only once before inoculation.

In this experiment, freeze-dried epidermal mucus powder of *E. fetida* was added to the culture medium to assess whether the *E. fetida* epidermal mucus would affect the extracellular enzyme activity of *B. bassiana*. The results showed that the enzymatic activities of the 4 extracellular enzymes studied significantly changed after treatment (Fig. 4). In the control group without *E. fetida* epidermal mucus (CK group), the fungal protease (Fig. 4A), lipase (Fig. 4B), chitinase (Fig. 4C), and *N*-acetyl-β-d-glucosidase (NAG) enzyme (Fig. 4D) activities in the medium all exhibited a similar trend that increased in the first phase and then decreased in the later phase.

In contrast, in the treatment group with *E. fetida* epidermal mucus added every day (EW group), the enzymatic activities of the subtilisin-like protease, lipase, chitinase and NAG remained at very low levels (Fig. 4A–D). The enzymatic activities of these 4 extracellular enzymes of *B. bassiana* were inhibited by the addition of epidermal mucus to the medium.

In treatment group, which contained *E. fetida* epidermal mucus added only once at inoculation (EO group), compared with the other two groups, the activities of 4 extracellular enzymes showed different trends. During days 1–4 after inoculation, the enzymatic activities of the 4 extracellular enzymes remained at very low levels (Fig. 4A–D). However, the activities of the 4 extracellular enzymes increased daily from the 4th day. On the 8th day, the activities of 4 extracellular enzymes increased significantly.

### The effect of *E. fetida* epidermal mucus on the pathogenicity of *B. bassiana*

**Figure 5:**
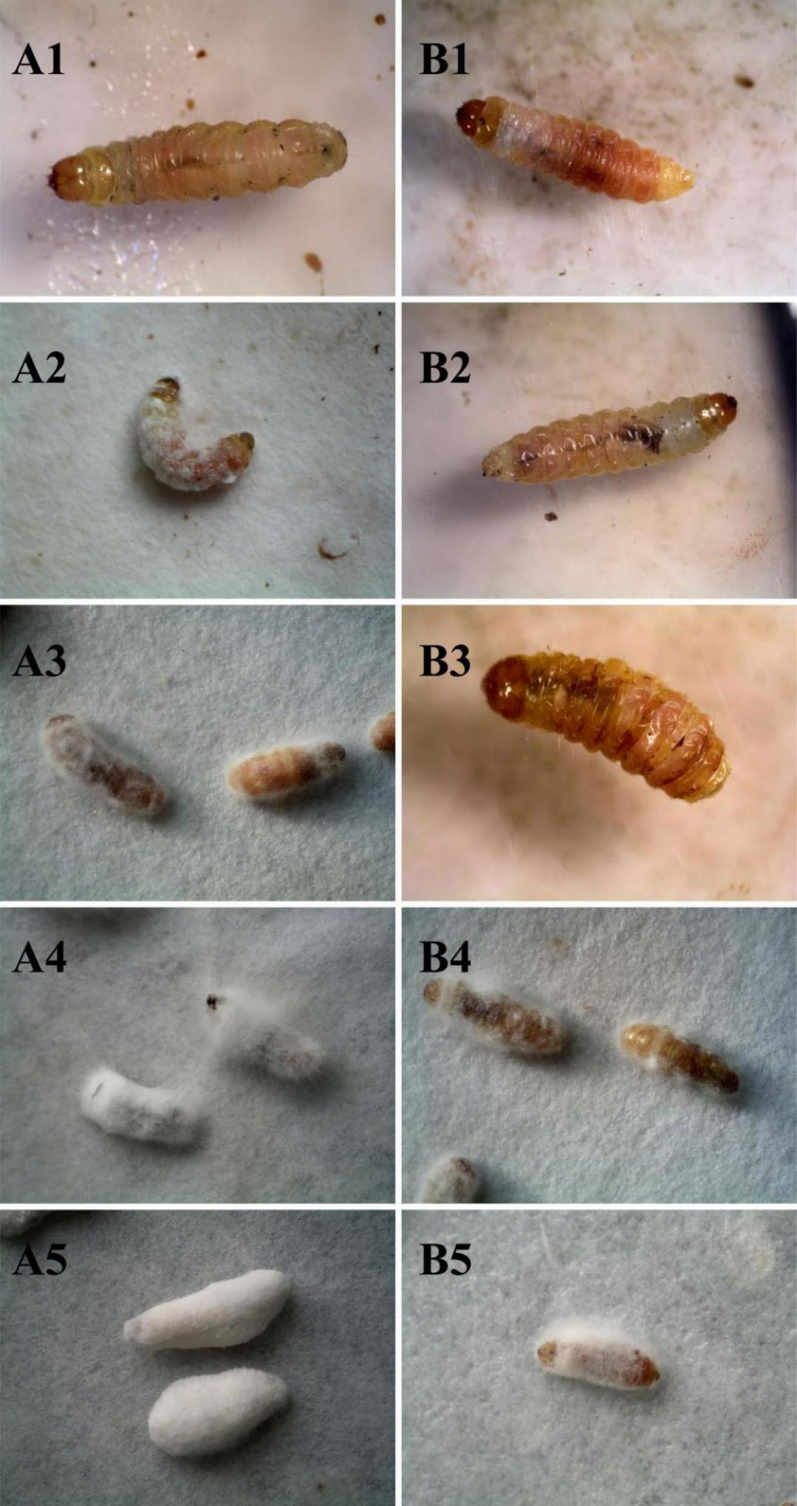
Comparison of the infective symptoms of *A. hetaohei* larvae infected by the *B. bassiana* treated or not treated with the epidermal mucus of the earthworm *E. foetida.* (**A**): treatment group 3, in which, the *B. bassiana* before inoculation, was not treated with the epidermal mucus of the earthworm *E. foetida*. (**A1–A5**) showing the infection symptoms of larvae on the 3rd, 5th, 7th, 10th and 14th days, respectively, after inoculated. (**B**): treatment group 4, in which, the *B. bassiana* before inoculation, was treated with the epidermal mucus of the earthworm *E. foetida*. (**B1–B5**) showing the infection symptoms of larvae on the 3rd, 5th, 7th, 10th and 14th days, respectively, after inoculated.

The results showed that in the treatment group with *B. bassiana* spores only, after inoculation, the *A. hetaohei* larvae slowly deepened their body color, and individual larvae began to die on the 3rd day (Fig. 5A1). White hyphae appeared on the surface of the larvae on the 5th day (Fig. 5A2). The dead worms became stiff and surrounded by white hyphae on the 7th day (Fig. 5A3). From 10 to 14th day, the larvae were completely covered with white hyphae and spores (Fig. 5A4,A5). In the treatment group with *B. bassiana* spore suspension supplemented and earthworm epidermal mucus, the larvae showed slow infection symptoms after inoculation. Their activity decreased on the 3rd day (Fig. 5B1). On the 5th day, the larvae showed symptoms of infection and lesions on the body surface (Fig. 5B2). On the 7th day, the infected larvae died one after another (Fig. 5B3). On the 10th day, white hyphae appeared successively on the surface of larvae (Fig. 5B4). On the 14th day, the larvae were completely surrounded by white hyphae (Fig. 5B5).

**Figure 6:**
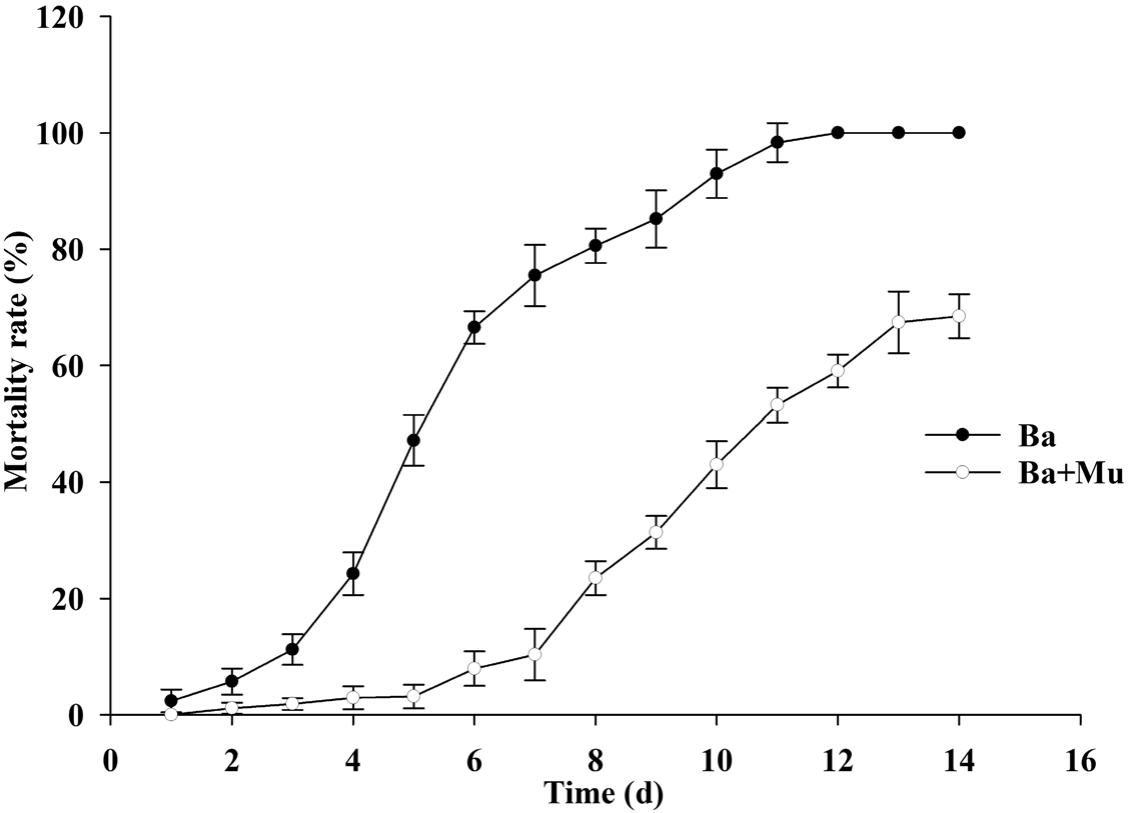
Comparison of the corrected mortality of *A. hetaohei* larvae infected by the *B. bassiana* treated or not treated with the epidermal mucus of the earthworm *E. foetida*. Ba: control group, the corrected mortality of *A. hetaohei* larvae were inoculated with the *B. bassiana* that was not treated with the earthworm epidermis mucus. Ba + Mu: the corrected mortality of *A. hetaohei* larvae were inoculated with the *B. bassiana* that was treated with the earthworm epidermis mucus.

After 14 days of continuous observation and mortality statistics, the treatment group with sterilized distilled water only was used to calculate the corrected mortality. The results showed that, there was no significant difference between the treatment group with sterilized distilled water and the treatment group with earthworm epidermal mucus. The mortality of the *A. hetaohei* larvae treated by *B. bassiana* spores with *E. fetida* epidermal mucus was relatively low in the first 5 days. At 3 d and 5 d, the cumulative corrected mortality of the larvae was 2.247 ± 1.619% and 4.560 ± 2.068%, respectively. In the control group without *E. fetida* epidermal mucus (CK group), the cumulative corrected mortality rate was 11.236 ± 2.570% and 47.126 ± 3.320%, respectively. After 5 d, the mortality rate of larvae in the treatment group increased significantly. At 7 d, 10 d, and 14 d, the cumulative corrected mortality of larvae was 10.588 ± 4.342%, 45.238 ± 4.025%, and 67.901 ± 3.782%, respectively, while that in the CK group was 74.118 ± 5.263%, 94.048 ± 4.166%, and 100%, respectively (Fig. 6). The mortality rate of larvae in the treated group was lower than that in the CK group, and the difference was significant. The median lethal time of the larvae in the treatment group of *B. bassiana* with *E. fetida* epidermal mucus was 10.664 d, but that in the CK group was 5.537 d. This difference reached significance. This experiment showed that the *E. fetida* epidermal mucus did have a certain influence on the pathogenicity of *B. bassiana* to the larvae in 2 ways: the mortality rate of larval was reduced and the lethal time of larvae was prolonged. Nevertheless, the surviving *B. bassiana* still maintain certain infectivity to the insect.

## Discussion

The strian *B. bassiana* is one of the most important entomopathogenic fungi and can persist in soil for many years^1,29^. Earthworms are important and abundant invertebrates in soil and interact closely with other organisms, especially microorganisms, in the soil^17,20,30–33^. In this paper, the effect of the *E. fetida* epidermal mucus on *B. bassiana* in fungal vitality and infectivity to insects was studied, and some novel results were obtained.

The main pathway by which *B. bassiana* infects insects is integument infection. The fungal spores must adhere to the cuticle of the insects, germinate, and produce germ tubes and hyphae; then, they penetrate into the insect integument and infect^3,34^. In this study, by using SEM, it was observed that the spores of *B. bassiana* may adhere to the epidermis of *E. fetida* and are often attached at the intersegmental furrows, annulus on the segments, and grid-like structures between the annuli and around the stomata. It is very similar to the characteristics of *B. bassiana* adhesion on insect cuticles^24^. However, the difference is that the adhered spores on the epidermis of the earthworm *E. fetida* did not germinate, but they could be covered by the earthworm epidermal mucus and change their shape from smooth and plump at first to rough and shrunken and even shriveled. This indicated that *B. bassiana* spores can be attached to the epidermis of earthworms, but it is hard for them to germinate on the epidermis of earthworms. It is speculated that the *E. fetida* epidermal mucus may play an antifungal role on *B. bassiana* and cause inactivation of *B. bassiana* spores.

To verify this conjecture, *E. fetida* epidermal mucus at five concentrations was used to treat spores of *B. bassiana,* and their effect on spore germination and colony growth was observed. In this experiment, about 10 mg of mucus lyophilized powder can be obtained per 1 mL of mucus. It is speculated that the natural concentration of earthworm mucus is about 10 mg/mL. Therefore, 20.00 mg/mL, 10.00 mg/mL, 5.00 mg/mL, 2.50 mg/mL, and 1.25 mg/mL mucus solution was used to observe the germination of *B. bassiana* spores. The results showed that the *E. fetida* epidermal mucus could significantly inhibit the spore germination of *B. bassiana* in a dose-dependent manner. It was reported that the *E. fetida* epidermal mucus is composed of carbohydrates, lipids, peptides, proteins, amino acids and other molecules^13,15,16,35^, and some mucopolysaccharide and protein components have antibacterial effects^16,36^. Wang^16,37^ isolated some antimicrobial peptides and polysaccharide from the surface mucus of earthworms and demonstrated that these antimicrobial peptides and lectins have obvious inhibitory effects on *Escherichia coli* and other bacteria. Thus, it is very likely that the inhibitory effect of the epidermal mucus of the earthworm *E. fetida* on *B. bassiana* is also from the related components of the antimicrobial peptides and some mucopolysaccharides. However, the compositions of the *E. fetida* epidermal mucus and their antimicrobial components remain to be further studied.

In the process of *B. bassiana* invading the insect cuticle, in addition to the mechanical pressure of fungal penetration to the insect cuticle, degradation to the insect cuticle by extracellular enzymes secreted by the fungus plays an important role^38,39^. This is because the cuticle of the insect body wall is composed of the epicuticle and protoepidermis (outer epidermis and inner epidermis), and the upper epidermis includes the wax protecting layer, wax layer and cuticle seminal layer. The insect epidermis consists of an epicuticle and procuticle. The epicuticle includes the cement layer, wax layer and cuticulin layer from external to internal, and the procuticle is mainly composed of chitin and protein. Additionally, extracellular protease, chitinase and lipase are secreted by *B. bassiana* to degrade protein, chitin and ester of the insect cuticle. The activity of extracellular enzymes of *B. bassiana* is usually considered an important index to detect its infectivity to host insects^40,41^. In fact, these four groups of extracellular enzymes secreted by *B. bassiana* belonging to extracellular proteases, chitinases and lipases which, are usually considered to be secreted by *B. bassiana* in degrading protein, chitin and ester of the insect cuticle. The exact number of enzymes in each of four groups could be further estimated by mining the known genomes of *B. bassiana* species. This problem may need further study.

In this study, *E. fetida* epidermal mucus lyophilized powder was added to liquid medium every day in the treatment group EW, and the *E. fetida* epidermal mucus lyophilized powder was only added once at the beginning of culture in the treatment group EO. It was found that during incubation of *B. bassiana*, the epidermal mucus of the earthworm *E. fetida* did have a significant inhibitive effect on the 4 fungal extracellular enzymes (subtilisin-like protease, lipase, chitinase, and NAG), and the inhibitive effect could remain for a period of time. Therefore, it is suggested that the epidermal mucus of *E. fetida* can inhibit *B. bassiana* not only for spore germination but also for extracellular enzyme secretion. However, if *B. bassiana* is no longer exposed to the newly secreted *E. fetida* epidermal mucus, the inhibition effect will gradually decrease.

Shapiro Ilan and Brown (2013) found that *B. bassiana* in soil in which the earthworm *Lumbricus terrestris* (L.) has been present could still infect *Galleria mellonella* (L.) larvae. However, the specific effect of the earthworm epidermal mucus on *B. bassiana* infection was not studied. To verify whether the *E. fetida* epidermal mucus had an effect on the pathogenicity of *B. bassiana*, the larvae of *A. hetaohei* were inoculated with *B. bassiana* spores that were treated with the *E. fetida* epidermal mucus in advance. The results showed that the *E. fetida* epidermal mucus could truly reduce the pathogenicity of *B. bassiana* to the insect. It could result in a slower disease course and a lower mortality of the larvae during infection. The practical data showed that within 5 d, the mortality rate of the larvae infected by *B. bassiana* in the group treated with *E. fetida* epidermal mucus was much lower than that of the CK group, but it increased rapidly after 5 d. The mortality rate of the treatment group increased from 3.45% on the 7th day to 41.67% on the 10th day , but it reached 91.67 ± 3.85% in the CK group. The mortality rate of the treatment group at 14 d was 65.43 ± 6.78%, while it reached 100% in the CK group. Moreover, the median lethal time of *B. bassiana* to the larvae in the group treated with the *E. fetida* epidermal mucus was 10.322 d in the treatment group, but it was 5.496 d in the CK group. Therefore, although the pathogenicity of *B. bassiana* to the insect *A. hetaohei* was affected by the epidermal mucus of the earthworm *E. fetida* and was reduced in infectivity to the insect, those *B. bassiana* spores that survived in the treatment with *E. fetida* epidermal mucus still maintained certain infectivity to the insect. In addition, the inhibitory effect of the *E. fetida* epidermal mucus on *B. bassiana* can gradually be relieved after 5–7 d with loss of the active component from the *E. fetida* epidermal mucus.

## Conclusions

As mentioned above, the experiment using the epidermal mucus of the earthworm *E. fetida* and fungus *B. bassiana* indicated the following conclusion: the epidermal mucus secreted by *E. fetida* has a significant inhibitory affect on the vitality and pathogenicity of *B. bassiana*. The inhibitive effect includes limiting fungal spore germination, reducing fungal extracellular enzyme secretion and activity, and weakening fungal infectivity and pathogenicity, which caused a slower infection and less mortality of the target insect. These inhibitory effects are interrelated with the concentration and extension time of the epidermal mucus of *E. fetida*. Nevertheless, those *B. bassiana* spores surviving after treatment with *E. fetida* epidermal mucus still maintain a certain infectivity to the target insect. When *B. bassiana* is used as a biological insecticide, the application methods include spraying, dusting or directly applying into the soil. Whichever method is used, it will eventually stay in the soil and contact with earthworms. Previous studies did not consider the effect of earthworm epidermal mucus on *B. bassiana*. Our study found that the earthworm epidermal mucus has a certain inhibitory effect on *B. bassiana*, including inhibiting spore germination and reducing the pathogenicity to insects. This finding is very important for applying *B. bassiana* in the biological control of insects. However, it is necessary to study the antifungal mechanism of the epidermal mucus of the earthworm *E. fetida* to *B. bassiana.*

Earthworms live in an environment with a variety of microorganisms. In order to cope with changing environmental conditions, earthworms require highly responsive stress mechanisms. It has been reported that the expression of immune-related earthworm cellulase in *Eisenia andrei* was up-regulated after treatment with *Bacillus subtilis* and *Escherichia coli*^42^. It was also reported that the expression of cellulase, Lumbrokinase, lysozyme, HSP70 and other immune-related proteins in *E. fetida* were up-regulated after treatment with *E. coli*^43^. Under the stimulation of *Saccharomyces cerevisiae*, the expression of pattern recognition receptor CCF (coelomic cytolytic factor) and lysozyme was up-regulated in *E. andrei*^44^. Earthworm epidermal mucus is the first defense line of its innate immunity. Earthworms epidermal will secrete a large amount of mucus in response to a stimulus, and form a mucus film wrapped in the surface of the earthworm, which has a protective effect on the earthworm^20,22^. However, it is not reported whether the genes related to innate immunity and mucus secretion of earthworms will change their expression after the earthworms are treated with *B. bassiana*. Earthworm epidermal mucus is also a complex of many components and the secretion of mucus should be involved by many genes, so it is a subject worthy of further study in the future.

## Materials and methods

### Earthworm, fungi and insects

Earthworm *E. fetida* were obtained from the earthworm breeding base in Baiyangdian, Hebei, China and raised in a laboratory at 20 °C with 70% soil humidity. Adult clitellate earthworms of similar body size were selected for this study.

The *B. bassiana* TST05 strain was cultivated in the laboratory at 25 °C with 75% relative humidity (RH), and fungal conidia were collected for the study. Two media were used in the experiments described below: (1) *B. bassiana* rejuvenation: Potatoes 200 g, Glucose 20 g (Tianjin FengChuan chemical reagent Technology Co., Ltd), Agar 20 g (Beijing solab Technology Co., Ltd), cuticle powder of target insects 5 g, Distilled water 1000 mL, pH value of the natural^45^. (2) Potato Dextroxa Agar (PDA): Potatoes 200 g, Glucose 20 g (Tianjin FengChuan chemical reagent Technology Co., Ltd), Agar 20 g (Beijing solab Technology Co., Ltd), Distilled water 1000 mL, pH value of the natural.

The mature larvae of *A. hetaohei* Yang (Lepidoptera: Heliodinidae) were selected as the target insects infected by *B. bassiana* strain TST05 in this study. Before the experiment, walnut worm fruit was collected in late August from an experimental walnut orchard in Yuxian County, Shanxi Province, China, and then, the walnut worm fruit was placed in the laboratory in the shade. When the mature larvae naturally drilled out of the fruit, they were collected and placed in a culture dish covered with a wet filter for infection study.

### SEM observation of the attachment and germination of *B. bassiana* on the earthworm epidermis

The *B. bassiana* spores were placed in sterilized distilled water containing 0.1% Tween 80 and the concentration was 1.0 × 10^7^ spores/mL. Spore concentration was counted under microscope by using cell counting plate. The immersion method was used for earthworm infection. Earthworm *E. fetida* sample individuals were steeped for 10 s into the spore suspension of *B. bassiana* and then maintained at a temperature of 15 ℃ for 10 min. Then, earthworms were transferred in a beaker (500 mL) lined with wet filter paper. Samples were collected at 1 h, 3 d and 7 d and fixed with 2.5% glutaraldehyde solution at 4 °C for 48 h. Then, the earthworm samples were rinsed thrice with 0.2 M phosphate buffer and incubated for 1.5 h in 1% (v/v) osmium tetroxide in the same buffer at 4 °C. Then, the samples were washed with ddH_2_O and dehydrated in a series of acetone solutions, which was replaced by liquid carbon dioxide. Finally, the samples were dried using an EMS 850 critical point drying apparatus (Electron Microscopy Sciences, Hatfield, Pennsylvania, USA) and treated with gold spray. The gold-sprayed samples were observed and photographed using a scanning electron microscope (SEM) (model JSM-840, JOL, Ltd., Japan).

### Detection of the effect of the *E. fetida *epidermal mucus on *B. bassiana* spore germination

The *E. fetida* individuals were stored in a sterile beaker (500 mL) with sterilized distilled water. After 30 min of incubation, the epidermal mucus secreted by the earthworms was collected with sterilized distilled water. Then, a filter membrane was used to remove the bacteria, and the water was removed from the epidermal mucus using a freeze-drying technique. The dry matter of the epidermal mucus of *E. fetida* was prepared into 1.25 mg/mL, 2.50 mg/mL, 5.00 mg/mL, 10.00 mg/mL, and 20.00 mg/mL mucus solutions using sterilized distilled water. Sterile distilled water was used instead of the earthworm epidermal mucus solution in the control group. The treatment of each concentration was repeated 3 times.

A suspension of *B. bassiana* spores (200 spores/mL) was mixed with different concentrations of the mucus solutions in a 1:9 ratio. After incubation at 4 °C for 2 h, 1 mL of the mixture was smeared onto PDA medium and incubated at 25 °C for 7 d. The fungal colonies of *B. bassiana* were observed and counted. The experiment was repeated 5 times in each group.

### Detection of the inhibitory effect of the *E. fetida* epidermal mucus on the extracellular enzyme activities of *B. bassiana*

Triangle bottles with 40 mL of the liquid medium (1.5 g/L KH_2_PO_4_, 0.6 g/L MgSO_4_, 0.5 g/L KCl, 1 mg/L FeSO_4_·7H_2_O, 1 mg/L ZnSO_4_·7H_2_O, and 7.5 g/L insect cuticle powder) were sterilized by autoclaving. Then, 1 mL of the spore suspension (10^8^ spores/mL) of *B. bassiana* and 9 mL of the epidermal mucus solution (2.5 mg/mL) of *E. fetida* were mixed and inoculated into the medium. Then, the triangle bottles were put into an oscillator and incubated at 25 °C for 8 d. During the culture process, the treatment group EW was supplemented with 10 mg earthworm mucus lyophilized powder every day, but the treatment group EO was no longer given epidermal mucus lyophilized powder. In the control group (CK), only spore suspension was added, and the earthworm epidermal mucus solution was replaced by sterile distilled water. The experiment was repeated 5 times in each group.

Preparation of crude enzyme solution: During cultivation, 300 μL of culture solution was collected every 24 h in each triangle bottle. The solution samples were centrifuged at 150,000*g* for 20 min, and the supernatant was the crude enzyme solution. The crude enzyme solution was stored at − 80 °C before use.

Subtilisin-like protease assay: The method reported by St. Leger was employed (1987)^46^. One unit of protease activity was defined as the amount of enzyme that catalyzes the decomposition of Suc-(Ala)_2_-Pro-Phe-pNA to produce 1 μg nitroaniline per minute.

Lipase assay: The method reported by Silva was employed^47^. One unit of lipase activity was defined as the amount of enzyme that catalyzes the decomposition of fat to produce 1 μg *p*-nitrophenol per minute.

Chitinase assay: Chitinase was determined according to the instructions of the Chitinase Kit (BC0825, Beijing Solarbo Science & Technology Co., Ltd.). One unit of chitinase activity was defined as the amount of enzyme that catalyzes the decomposition of chitin to 1 μmol *N*-acetylglucosamine per hour.

NAG assay: NAG was determined according to the instructions of the β-*N*-acetylglucosaminidase Kit (A031-1-1 Nanjing Jiancheng Bioengineering Institute). One unit of NAG activity was defined as the amount of enzyme that catalyzes the hydrolysis of 1 μmol substrate per minute.

### Effect of earthworm epidermal mucus on the pathogenicity of *B. bassiana*

Four treatments were set up in the experiment: (1) sterilized distilled water only; (2) sterilized distilled water mixed with earthworm epidermal mucus (2.5 mg/mL) in a 1:9 ratio; (3) a suspension of *B. bassiana* spores (10^7^ spores/mL); (4) a suspension of *B. bassiana* spores (10^8^ spores/mL) mixed with earthworm epidermal mucus (2.5 mg/mL) in a 1:9 ratio. Each treatment was repeated 5 times.

The larvae of *A. hetaohei* were employed as infection insects. The larvae were soaked in the mixture or sterilized distilled water for 5 s and then placed in a Petri dish lined with wet filter paper and absorbent cotton at the bottom. Forty larvae were placed in each Petri dish, and each experiment was repeated 3 times. The larvae were placed in a 25 °C incubator, the activity and surface characteristics of larvae were observed every day, and the quantity of dead larvae was counted. After 15 days of observation, the corrected mortality was calculated.

### Statistical analyses

Data were analyzed using the SPSS 21.0 statistical software package. ANOVA and Tukey's HSD were performed for statistical analyses.
